# Continuous Gum Work

**Published:** 1869-03

**Authors:** 


### Editorial.

### CONTINUOUS GUM WORK.

We publish in this number of the Register an interesting paper on the subject of continuous gum work, by Dr. John Allen.

This style of work was introduced to the profession some fifteen years ago, mainly by Dr. Allen's efforts. Its use was adopted by a considerable number of the profession within a few years after its introduction, but with by no means uniform results; a few failing in their first efforts, abandoned it; others obtaining better results, but not meeting with entire satisfaction gave it up, after a longer or shorter period. Within the last seven or eight years, there has been very little of it done; indeed, with here and there an exception, none at all.

A review of the causes for the non-adoption of this style of work, by the profession generally, may perhaps not be without interest.

First and negatively, it was not because of any failure in the method to answer the indications and requirements of an artificial denture; for this, all who are familiar with it will acknowledge that it possesses in the highest degree, or at least to a greater extent than any other method hitherto employed.

By that process, with the materials and teeth now furnished, artificial dentures can be made, by proper skill, that will defy detection under any casual observation. By it a far more perfect arrangement of teeth can be effected than by any other, both in respect to appearance and occlusion.

The deformities occasioned to the contiguous parts by the loss of the teeth, are more perfectly remedied by this kind of work, when properly made, than by any other. Its adaptation may be as thoroughly and as easily made, as is that of anything else.

The question still occurs, if these things are so, why is not this work in general use? In reply to which we will say, that it requires for its production a higher type of skill, than any other method. In what respects? it may be asked. Perhaps no more skill in procuring an impression, in making models and counter-models, in forming and swaging the plate, is required than for making one of gold, yet each of these steps must be very skill-fully and faithfully performed, to secure the best results. The bite or articulation is obtained upon the same general principle as for other work. But now comes the point, where high artistic skill is required, viz.: the selection and arrangement of teeth; and here we will be pardoned for making the suggestion, that a far greater variety of these are needed to meet the variety of cases presented; as every face has its individuality, so has every set of teeth; and the make up of this individuality, embraces form, shape, size, color and arrangement, and every case presented, requires something different from every other case. But how often do we see the Dentist buy, perhaps, fifty sets of teeth, nearly all from the same moulds, and varying but little in color, and yet take them up almost in rotation, and thrust them into the mouth of whoever may present themselves, man, woman, or child; and with such skill, of course, no attempt whatever is made for the restoration of deformed parts; and hence it is, that we so frequently see those Dental caricatures (monstrosities we had almost said,) in the mouths of faces otherwise beautiful.

In the arrangement of continuous gum teeth, any variation or arrangement that may be desired is attainable, which is not true of any other mode, except "Rose Pearl." The teeth being separate one from another, may each have one inclination or another, shortened or lengthened, one or more turned upon its axis; all this, and more, just at the option of the operator.

The attention and study, necessary for the highest results, in this particular is not, and will not be exercised by a very large majority of our profession. There are various reasons for this, among which are the facts, that very many have not the natural ability, and have not the susceptibility of being educated to any considerable extent; and others, are situated in communities that have so low an appreciation of the beautiful, that no encouragement would be extended to any one, who should essay its production.

In the arrangement of artificial teeth, the only safe course for any one possessed of a low degree of skill, is to make as perfectly uniform regularity as possible; if such an one attempts irregularity in the arrangement, he is liable to the grossest blunders, giving perhaps to the round, regular, smooth-featured face, teeth quite irregular in arrangement, while to the face, irregular, angular in form and expression, he would place the teeth in perfect circle, as trim as well drilled soldiers.

In the use of teeth in mastication, very much depends upon the occlusion of the upper and lower set; this can be most perfectly accomplished in continuous gum work.

The next point requiring special attention is putting on and carving the body. This over the main plate, should be of as nearly uniform thickness as possible. Close attention and experience will enable almost any one to obtain good results in this respect.

One of the greatest difficulties in the way of the general introduction of this style of work, is the management of the furnace and baking; in this every one must learn by experience, and there are some, that experience, though a good schoolmaster in general, fails to bring up to any high degree of attainment.

In order to be an adept in the use of the furnace, one should be constantly engaged at it. Much depends upon the furnace, its form, situation, etc. Nearly all the furnaces that have been in use, in the West at least, have, as we know from experience, been very deficient in form and structure.

Another source of failure has been in the fuel employed, coke has been almost the only fuel used in Dental furnaces heretofore, and many failures occur from that source alone. A heat from it is very fluctuating, changing almost constantly; it oftentimes contains a large amount of sulphur and other deleterious agents, that are destructive to porcelain; it destroys muffles very rapidly. Anthracite coal is the only fuel that should be used in a porcelain furnace; it is free from injurious substances, makes a uniform and beautiful heat. A fire of this coal, well prepared, may be brought up to the requisite degree, and by proper management retained at that for hours. With such an arrangement of furnace and fire, the baking of continuous gum work becomes an easy and certain process.

We are glad to see that attention is being directed to this work. Some are taking it up for the first time, others are returning to it, who some years ago abandoned it, being seduced therefrom by the fatal delusion that something easier to work, for the Dentist, and cheaper for the people was required. It was a delusion, the utter mischievousness of which almost every one is free to confess, yet while so confessing, there are some who clasp it to their bosom, while it stings their profession to its core. T.

