# Correction

**Published:** 1869-08

**Authors:** G. W. Dunn


### CORRECTION.

Mr. Editor.—In the March number of the Dental Register, page 136, is the following, viz: "In the arrangement of continuous gum teeth, any variation or arrangement that may be desired is attainable, which is not true of any other mode except 'Rose Pearl.'" This exception though perhaps true as far as the experience of the writer goes, should have included the mineral plate and teeth combined, which certainly possess superior advantages in this respect, as now manufactured under Dunn's improved method. Permit me to ask a correction of this, as in your judgment may be most proper.

Respectfully Yours,

G. W. Dunn.

